# RAGE: A potential therapeutic target during FGF1 treatment of diabetes‐mediated liver injury

**DOI:** 10.1111/jcmm.16446

**Published:** 2021-03-31

**Authors:** Peipei Zheng, Zonghao Tang, Jun Xiong, Beini Wang, Jingyu Xu, Lulu Chen, Shufang Cai, Chengbiao Wu, Libing Ye, Ke Xu, Zimiao Chen, Yanqing Wu, Jian Xiao

**Affiliations:** ^1^ Department of Endocrinology The First Affiliated Hospital and School of Pharmaceutical Sciences Wenzhou Medical University Wenzhou China; ^2^ The Institute of Life Sciences Wenzhou University Wenzhou China; ^3^ Key Laboratory of Medical Electrophysiology of Ministry of Education Drug Discovery Research Center Southwest Medical University Luzhou China; ^4^ Clinical Research Center Affiliated Xiangshan Hospital Wenzhou Medical University Wenzhou China

**Keywords:** apoptosis, diabetes‐mediated liver damage, fibroblast growth factor 1, inflammation, receptor of advanced glycation end products

## Abstract

As a serious metabolic disease, diabetes causes series of complications that seriously endanger human health. The liver is a key organ for metabolizing glucose and lipids, which substantially contributes to the development of insulin resistance and type 2 diabetes mellitus (T2DM). Exogenous fibroblast growth factor 1 (FGF1) has a great potential for the treatment of diabetes. Receptor of advanced glycation end products (RAGE) is a receptor for advanced glycation end products that involved in the development of diabetes‐triggered complications. Previous study has demonstrated that FGF1 significantly ameliorates diabetes‐mediated liver damage (DMLD). However, whether RAGE is involved in this process is still unknown. In this study, we intraperitoneally injected db/db mice with 0.5 mg/kg FGF1. We confirmed that FGF1 treatment not only significantly ameliorates diabetes‐induced elevated apoptosis in the liver, but also attenuates diabetes‐induced inflammation, then contributes to ameliorate liver dysfunction. Moreover, we found that diabetes triggers the elevated RAGE in hepatocytes, and FGF1 treatment blocks it, suggesting that RAGE may be a key target during FGF1 treatment of diabetes‐induced liver injury. Thus, we further confirmed the role of RAGE in FGF1 treatment of AML12 cells under high glucose condition. We found that D‐ribose, a RAGE agonist, reverses the protective role of FGF1 in AML12 cells. These findings suggest that FGF1 ameliorates diabetes‐induced hepatocyte apoptosis and elevated inflammation via suppressing RAGE pathway. These results suggest that RAGE may be a potential therapeutic target for the treatment of DMLD.

## 1 INTRODUCTION

Two‐thirds of type 2 diabetes (T2DM) suffer from non‐alcoholic fatty liver (NAFLD).^1^, ^2^ Diabetes promotes the development of NAFLD through inducing necrotic inflammation and fibrosis progression, especially the development of non‐alcoholic steatohepatitis (NASH).^3^ Elevated inflammation is an important molecular mechanism underlying liver injury. Recently, litter is known about the mechanism underlying diabetes‐accelerated liver necrosis inflammation and fibrosis. With the continuous increase in the global incidence of T2DM and NAFLD, it is of great significance to further explore the molecular mechanisms and potential treatment strategies of diabetes‐mediated liver damage (DMLD).

RAGE is a receptor for advanced glycation end products and a member of the transmembrane receptor of the immunoglobulin superfamily. RAGE has been reported to be involved in the activation of multiple intracellular signalling pathways, such as nicotinamide adenine dinucleotide phosphate (NADPH) enzyme, proteinase C and MAPKs, thereby participating in cellular inflammation.^4^ Additionally, RAGE can participate in cell autophagy, proliferation and apoptosis by regulating PI3K/AKT and JAT‐STAT signalling pathways.^5^, ^6^ Moreover, RAGE is also considered as an important mechanism under diabetes‐associated complications. It has been reported that RAGE plays an important role in diabetic dementia, which is a pathological mechanism linking diabetes and Alzheimer's disease.^7^, ^8^ At the same time, RAGE has been reported to promote the progress of diabetic atherosclerosis, diabetic nephropathy and diabetic retinopathy.^9^, ^10^, ^11^, ^12^ Here, we speculate that RAGE may be also a key target during DMLD.

Fibroblast growth factor (FGF1) is an autocrine/paracrine regulator that acts on cells in various tissues. As an insulin sensitizer, FGF1 stimulates glucose uptake in an insulin‐dependent manner^13^ and then regulates the steady‐state control of euglycaemia.^14^ Except for controlling the blood glucose, FGF1 also ameliorates many diabetes‐induced complications through multiple signalling pathways. Studies in recent years have reported that FGF1 inhibits inflammatory level, then ameliorates diabetic nephropathy and improves insulin resistance through regulating JNK pathway.^15^, ^16^ Furthermore, FGF1 has been reported to block cellular stress and autophagy, and consequently ameliorates diabetes‐mediated hepatocyte apoptosis.^17^ However, the specific target of FGF1 during improving DMLD is still unclear.

In this study, we used db/db mice as an experimental diabetic mouse model to investigate whether RAGE is a key molecular target during FGF1 treatment of DMLD. We found that FGF1 ameliorates diabetes‐induced hepatocyte apoptosis and elevated inflammation via suppressing RAGE expression, suggesting RAGE may be a potential therapeutic target for the treatment of DMLD.

## 2 MATERIALS AND METHODS

### 2.1 Animal experiments

A total of 30 healthy 12‐week‐old male db/db (C57BLKS/J‐leprdb/leprdb) mice and 15 of their non‐diabetic db/m litters were purchased from the Model Animal Research Center of the Nanjing University, Nanjing City, Jiangsu Province. All experimental procedures were performed in accordance with the National Institutes of Health Animal Care and Use Guidelines and have been approved by the Animal Care and Use Committee of the Wenzhou Medical University. Thereafter, the animals were carefully maintained under a 14‐hour light/10‐hour dark condition, and the mice were given free access to water and food. The db/db mice and db/m mice were given a standard mouse diet. The db/db mice were divided into two groups (15 per group) and intraperitoneally (ip) injected either with FGF1 (0.5 mg/kg body weight) or with physiologic saline every other day for 4 weeks. After treating with FGF1 for 4 weeks, the animals were anaesthetized with an overdose of 4% chloral hydrate. Finally, the serum and liver tissues of mice from different groups were taken for biochemistry and molecular biological analysis.

### 2.2 Biochemical analysis

After the mice were killed, their serum samples were harvested and processed for the following studies. To measure the function of liver under diabetes condition, the alanine aminotransferase (ALT) and aspartate aminotransferase (AST) levels in serum were estimated by Micro Glutamic‐Pyruvic Transaminase Assay Kit (Solarbio, BC1555) and Micro Glutamic‐Oxalacetic Transaminase Assay Kit (Solarbio, BC1565) according to the manufacturer's instructions.

### 2.3 H&E staining, PAS staining and Masson's staining

For H&E staining, the 5‐μm sections were dewaxed and hydrated, then stained with haematoxylin and eosin (H&E) reagents. It was applied to evaluate the histological changes in liver tissues. For PAS staining and Masson's staining, after the 5‐μm sections were dewaxed, Glycogen Periodic Acid‐Schiff Stain Kit (Solarbio, G1281) and Masson's Trichrome Stain Kit (Solarbio, G1340) were used to stain the liver tissues according to the instructions to evaluate the levels of glycogen and collagen in the liver tissues. Then, the images were observed and captured using a Nikon ECLIPSE 80i microscope (Nikon, Japan).

### 2.4 Immunofluorescence staining

For the liver tissue, the 5‐μm sections were dewaxed and then carefully permeated in 0.1% Triton X‐100 for 3 minutes at room temperature. Then, they were blocked with 5% BSA for 1h at 37°C. For AML12 cells, the cells were grown in 12‐well plate. After washing with PBS and fixing with chilled methanol for 20 minutes, the cells were blocked with 5% BSA for 1 hour at 37°C. The liver tissue sections and AML12 cells were incubated with the following primary antibodies at 4°C overnight: cleaved caspase‐3 (1:500; Cell Signaling Technology, 9664S) and RAGE (1:500; Santa Cruz, sc‐365154). After washing in PBS at 37°C for 3 times, the sections were once again incubated with Alexa Fluor 647 (1:1000; Abcam, ab150115) or Alexa Fluor 488 (1:1000; Abcam, ab150077) as secondary antibody for 2 hours and stained with DAPI solution for 5 minutes. Fluorescence images were captured using a Nikon ECLIPSE 80i microscope (Nikon).

### 2.5 AML12 cell culture and treatment

AML12 cells were purchased from the Cell Storage Center of the Wuhan University (Wuhan, China). AML12 cells were cultured in Dulbecco's modified Eagle's medium (DMEM/F12, Gibco) supplemented with 10% foetal bovine serum (FBS, Gibco) and 1% antibiotics (100 units/mL penicillin, 100 μg/mL streptomycin). They were incubated in a humidified atmosphere containing 5% CO_2_ at 37°C. 30 mmol/L glucose was added as the high glucose (HG) group. Then, 25 mmol/L D‐ribose, a RAGE agonist (MCE, HY‐W018772), was chosen to enhance RAGE activity of AML12 cells. The cells were pre‐treated with 100 ng/mL of FGF1 for 1 hour prior to treatment with 25 mmol/L D‐ribose or 30 mmol/L glucose. Thus, the cells were divided into four groups: control group, HG group, HG + FGF1 group and HG+FGF1+D‐ribose group.

### 2.6 Western blot analysis

In order to analyse the protein levels in the liver and cultured cells, the liver tissues and cells were sonicated in ice‐cold lysis buffer and centrifuged at 14 000 g for 15 minutes. Then, the supernatant was taken, and the protein concentration was determined by the Bradford method. After the lysate was mixed with loading buffer, heated at 100°C for 10 minutes, the protein was separated on a 10% or 12% gel, and transferred to a PVDF membrane (Bio‐Rad, Hercules, CA, USA). The membranes were blocked with 5% milk in TBST (TBS containing 0.05% Tween‐20) for 1.5 hours and incubated with the following primary antibodies for overnight at 4°C: Bax (1:1000; Cell Signaling Technology, 5023S), Bcl2 (1:1000; Cell Signaling Technology, 3498S), IL‐10 (1:1000; Santa Cruz, sc‐365858), IL‐6 (1:1000; Santa Cruz, sc‐57315), NF‐κBp65(1:1000; Cell Signaling Technology, 8242S), p‐NF‐κBp65 (1:1000; Cell Signaling Technology, 3033S) and GAPDH (1:1000; Proteintech, 60004‐1‐lg). After washing 3 times with TBST, the membranes were treated with secondary antibody (1:10 000; Multi Science, 70‐GAM007 or 70‐GAR001) for 2 hours at room temperature. The signals were captured under a chemiluminescent diode X‐ray imaging system (Bio‐Rad). All experiments were repeated three times with independently prepared tissues. And the experimental results are statistically analysed.

### 2.7 Enzyme‐linked immunosorbent assay (ELISA)

The ELISA was used to measure the level of inflammatory factor (IL‐6, IL‐1β and IL‐10) in serums and AML12 cells. For cells study, after pre‐treating with HG with or without FGF1, then the medium of AML‐12 cells was collected from cells. The levels of IL‐6 and IL‐10 were estimated in AML‐12 cells and serum using IL‐6 (Multi Science, 70‐EK206), IL‐1β (Multi Science, 70‐EK21B) or IL‐10 ELISA kits (Multi Science, 70‐EK210) following the manufacturer's instruction.

### 2.8 Statistical analysis

Each experiment was repeated at least three times. All substantive data used for statistical analysis are usually considered as mean ± SEM. GraphPad Prism 5 (USA) was used to carefully compare the experimental results through one‐way analysis of variance and Tukey's post hoc test. Statistical significance was accepted when *P* < .05.

## 3 RESULTS

### 3.1 FGF1 treatment suppresses diabetes‐induced liver dysfunction via inhibiting elevated fibrosis and steatosis in the liver

**FIGURE 1:**
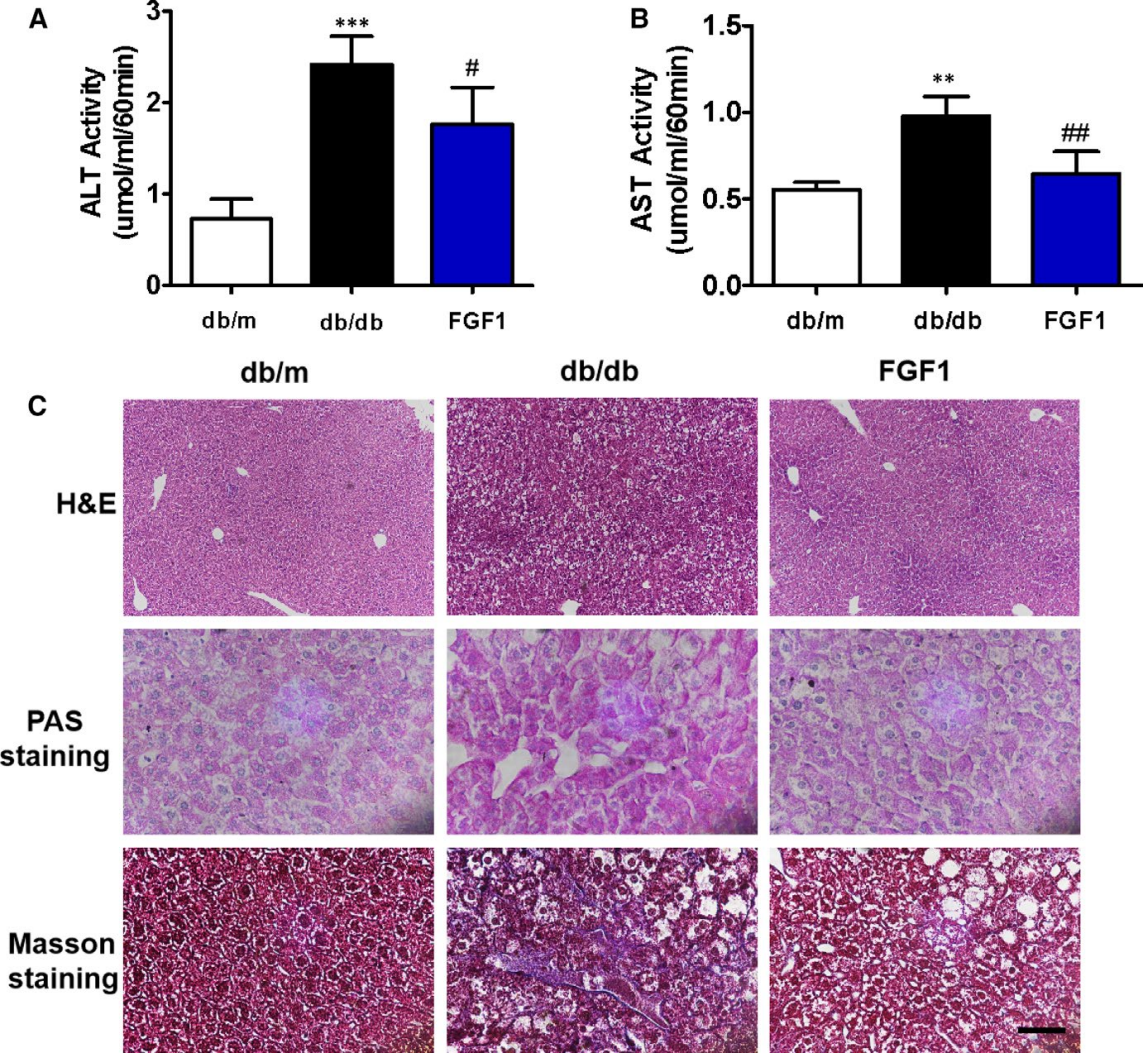
FGF1 treatment suppresses diabetes‐induced liver dysfunction via inhibiting fibrosis and steatosis in the liver. A, Plasma alanine aminotransaminase (ALT) level in different groups, n = 3. B, Plasma aspartate aminotransferase (AST) level in different groups, n = 3. C, Representative images of liver tissue stained with H&E staining (scale bars = 100 μm), PAS staining for evaluation of glycogen (scale bars = 15 μm) and Masson's trichrome staining for collagen (scale bars = 25um), n = 5. **P < .01, ***P < .001 vs db/m group; ^#^ *P* < .05, ^##^ *P* < .01 vs db/db group

Alanine aminotransferase (ALT) and aspartate aminotransferase (AST) are two common indicators of liver function.^18^ In our current study, it can be clearly shown that the ALT and AST levels in the serum of db/db mice are higher than those in the control group (Figure 1A,B). FGF1 administration significantly reduced the levels of ALT and AST in the serum of db/db mice (Figure 1A,B). Then, we explored the morphological changes in liver in db/db mice. As shown in H&E staining, the liver of db/db mice showed remarkable steatosis when compared to that of db/m mice and FGF1‐treated mice (Figure 1C). The PAS staining results indicated that the glycogen level in the liver of db/db mice is significantly higher than that in the liver of db/m mice and FGF1‐treated mice (Figure 1C). In addition, the content of collagen in the liver of db/db mice was significantly higher than that in the liver of db/m and FGF1‐treated mice, suggesting that the liver of db/db mice has developed severe fibrosis (Figure 1C). These results confirm that FGF1 treatment reversed diabetes‐induced liver dysfunction.

### 3.2 FGF1 treatment reduces diabetes‐mediated excessive cell apoptosis in the liver

**FIGURE 2:**
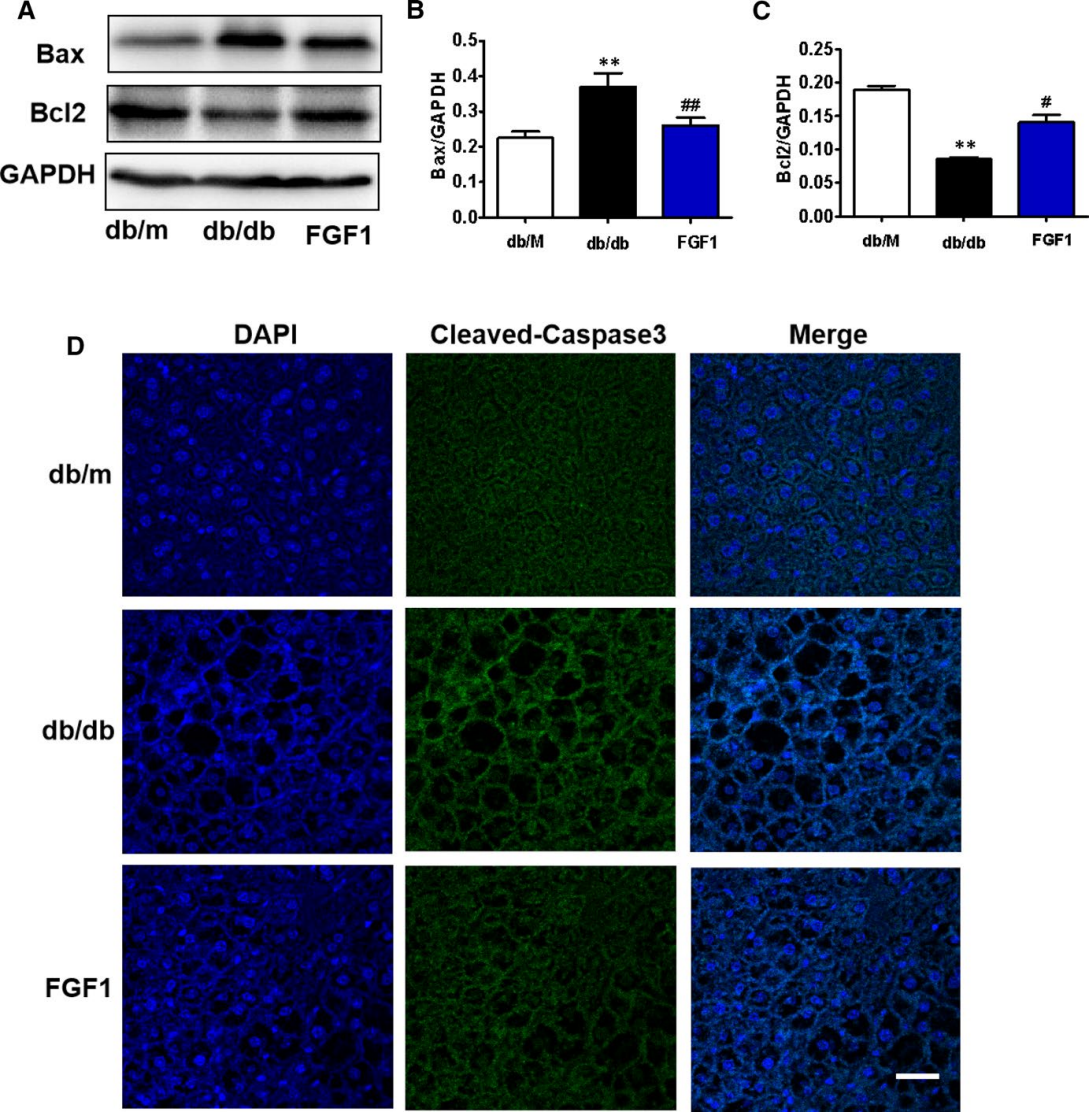
FGF1 treatment reduces diabetes‐induced excessive cell apoptosis in the liver. A, Western blotting results show the expression levels of Bax and Bcl2 in the liver from different groups. (B and C) Quantitative analysis of Bcl2 and Bax in the liver, n = 3. D, The immunofluorescence staining of cleaved caspase‐3 in the liver from different groups (scale bars = 25 μm), n = 5. **P < .01 vs db/m group; ^#^ *P* < .05, ^##^ *P* < .01 vs db/db group

Excessive apoptosis is a major cause factor for organ damage caused by diabetes.^19^, ^20^ Here, we have further investigated the role of apoptosis during DMLD development. Western blot results showed that diabetes significantly induces Bax overexpression and decreases Bcl2 expression in the liver (Figure 2A‐C). There was no significant difference in these two protein expressions between the FGF1‐treated group and the control group (Figure 2A‐C). Furthermore, we found that the fluorescence intensity of cleaved caspase‐3 is remarkably increased in hepatocytes from db/db mice when compared to that in the control group and FGF1‐treated group (Figure 2D).

### 3.3 FGF1 treatment ameliorates diabetes‐associated excessive inflammation in the liver

**FIGURE 3:**
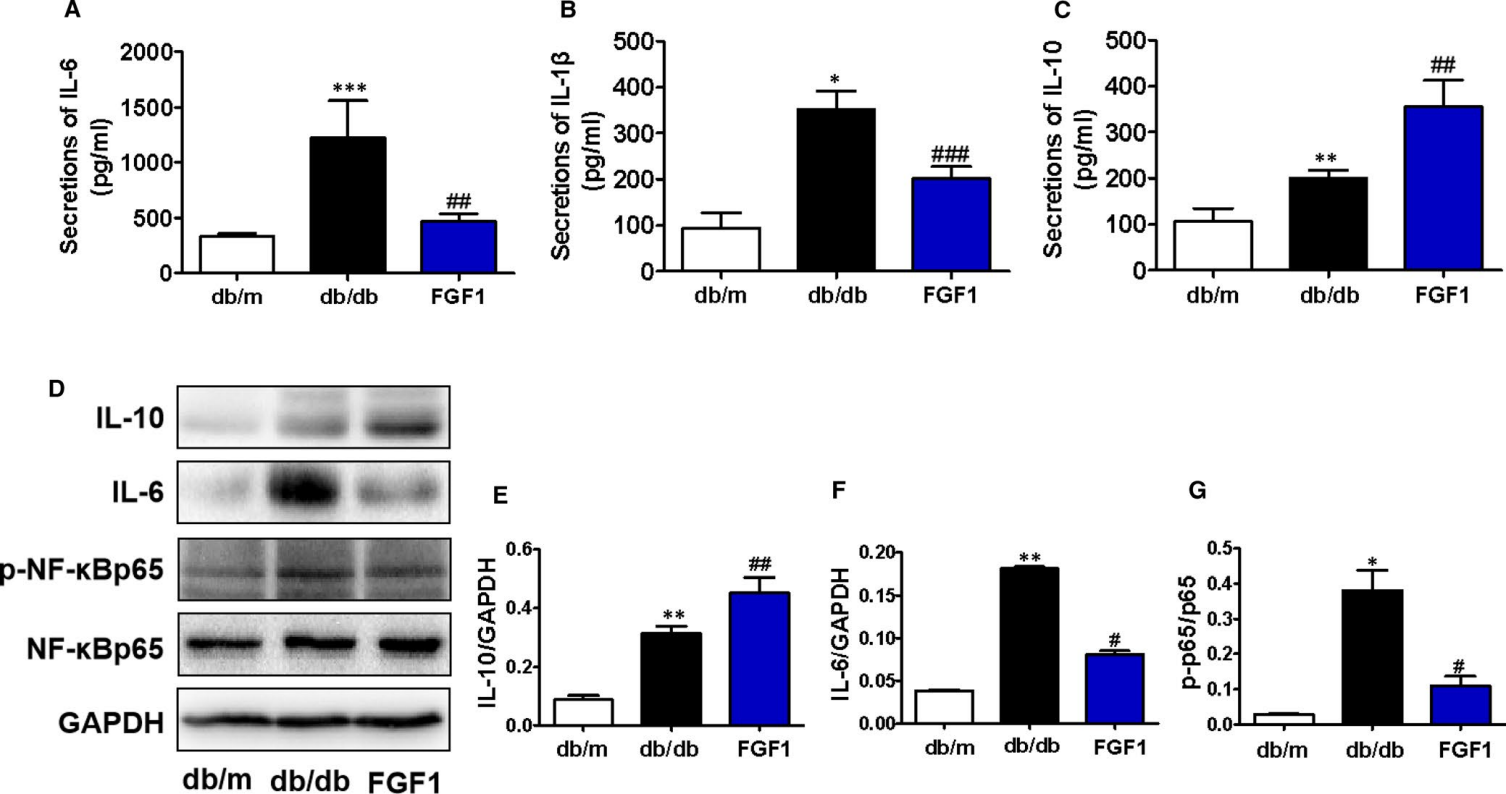
FGF1 treatment ameliorates diabetes‐associated excessive inflammation in the liver. (A‐C) Plasma concentrations of pro‐inflammatory cytokines (IL‐6 and IL‐1β) and anti‐inflammatory cytokine (IL‐10) in db/m, db/db and db/db + FGF1 groups, n = 5. (D‐G) The Western blotting and quantitative analyses of IL‐10, IL‐6, p‐NF‐κBp65 and NF‐κBp65 expressions in the liver from different groups, n = 3. *P < .05, **P < .01, ***P < .001 vs db/m group; ^#^ *P* < .05, ^##^ *P* < .01, ^###^ *P* < .001 vs db/db group

Chronic inflammation is essential for the fibrosis of liver tissue.^21^, ^22^, ^23^ In our current study, we tested whether FGF1 ameliorates the inflammation in the liver during DMLD. We measured the levels of pro‐inflammatory factors (IL‐6 and IL‐1β) and anti‐inflammatory factor (IL‐10) in the serum of mice (Figure 3A‐C). We found that diabetes significantly increases pro‐inflammatory factor levels (IL‐6 and IL‐1β), and FGF1 treatment remarkably reversed them (Figure 3A,B). However, diabetes also increased anti‐inflammatory factor level (IL‐10), and FGF1 treatment further enhanced the level of IL‐10 (Figure 3C). Moreover, we further detected the protein levels of p‐NF‐κBp65, IL‐10 and IL‐6 in the liver of mice (Figure 3D‐G). Consistent with the ELISA results, FGF1 treatment ameliorated diabetes‐induced elevated pro‐inflammatory factors (p‐NF‐κBp65 and IL‐6) and further enhanced the increase in IL‐10 in the liver of db/db mice (Figure 3D‐G).

### 3.4 FGF1 attenuates diabetes‐triggered elevated RAGE expression in the liver

**FIGURE 4:**
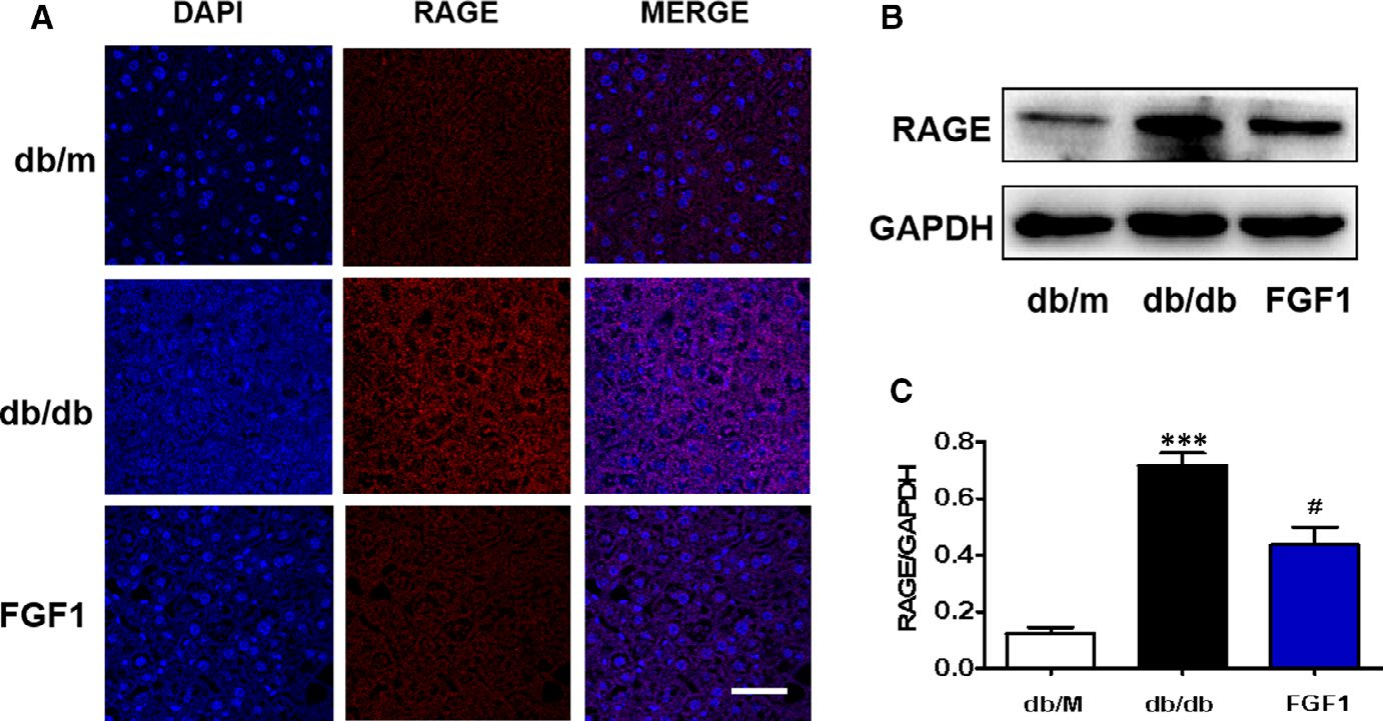
FGF1 attenuates diabetes‐triggered elevated RAGE expression in the liver. A, The immunofluorescence staining of RAGE in the liver from different groups (scale bars = 20 μm), n = 5. (B‐C) The Western blotting and quantitative analyses of RAGE expression in the liver from different groups, n = 3. ***P < .001 vs db/m group; ^#^ *P* < .05 vs db/db group

RAGE has been widely reported to be involved in diabetes‐associated complications,^8^, ^24^, ^25^ which is essential for many cellular processes, including inflammation, oxidative stress, proliferation and apoptosis.^4^, ^5^, ^6^ In order to detect whether RAGE participates in FGF1 during treatment of DMLD, we tested the expression level of RAGE in the liver of mice. Using immunofluorescence staining and Western blotting, we found that RAGE level in the liver of db/db mice is higher than that in the control group, and FGF1 treatment reverses it (Figure 4A‐C).

### 3.5 D‐ribose, a RAGE agonist, reverses the protective role of FGF1 during high glucose–induced excessive apoptosis in AML12 cells

**FIGURE 5:**
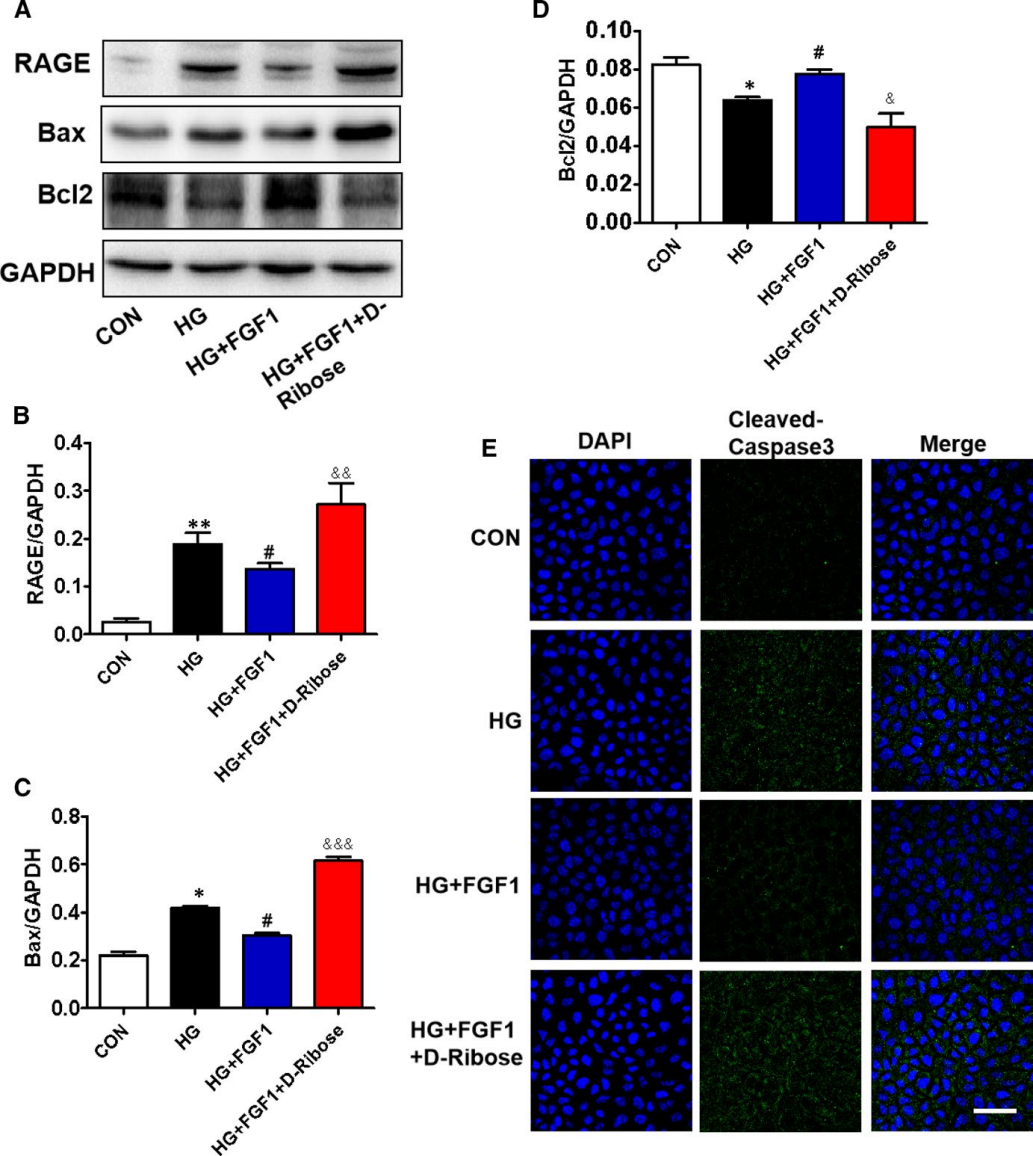
D‐ribose, a RAGE agonist, reverses the protective role of FGF1 during high glucose–induced excessive apoptosis in AML12 cells. (A‐D) The Western blotting and quantitative analysis of RAGE, Bax and Bcl2 expressions in AML‐12 cells from different groups, n = 3. E, The immunofluorescence staining of cleaved caspase‐3 in AML12 cells from different groups (scale bars = 50 μm), n = 5. *P < .05, **P < .01 vs CON group; ^#^ *P* < .05 vs HG group; ^&^ *P* < .05, ^&&^ *P* < .01, ^&&&^ *P* < .001 vs HG+FGF1 group

To further investigate the role of RAGE during FGF1 treatment of DMLD, D‐ribose (a RAGE agonist) was used to treat AML12 cells under high glucose condition. In our current study, we found that FGF1 treatment ameliorates high glucose‐induced up‐regulation of RAGE in AML12 cells, and D‐ribose treatment reverses it (Figure 5A,B). Moreover, high glucose induced overexpression of Bax and cleaved caspase‐3 and inhibited Bcl2 expression in AML12 cells (Figure 5A,C‐E). FGF1 treatment significantly ameliorated them (Figure 5A,C‐E). More importantly, D‐ribose treatment reversed the anti‐apoptotic effect of FGF1 by promoting RAGE expression (Figure 5A,C‐E). In summary, RAGE may be a key target for FGF1 exerting its anti‐apoptotic effect during DMLD.

### 3.6 D‐ribose blocks the role of FGF1 on high glucose–associated excessive inflammation in AML12 cells

**FIGURE 6:**
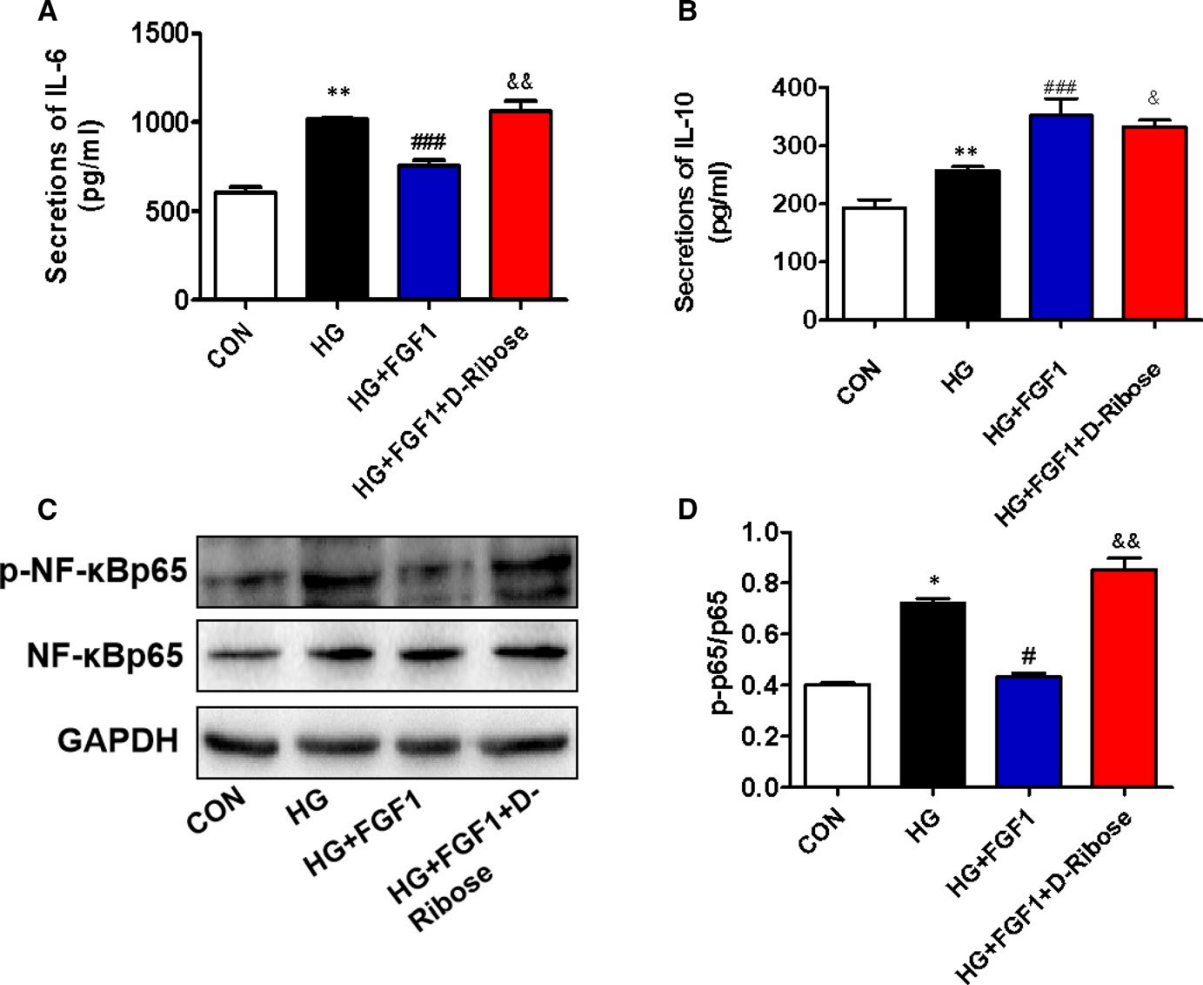
D‐ribose blocks the role of FGF1 on high glucose–associated excessive inflammation in AML12 cells. (A and B) The enzyme‐linked immunosorbent assay (ELISA) shows the levels of pro‐inflammatory cytokines (IL‐6) and anti‐inflammatory cytokines (IL‐10) in AML12 cells from different groups, n = 3. C, The Western blotting and quantitative analyses of p‐NF‐κBp65 and NF‐κBp65 in AML‐12 cells from different groups, n = 3. *P < .05, **P < .01 vs CON group; ^#^ *P* < .05, ^###^ *P* < .001 vs HG group; ^&^ *P* < .05, ^&&^ *P* < .01 vs HG+FGF1 group

We have further explored the relationship between RAGE and anti‐inflammatory function during FGF1 treatment of DMLD. Compared with the control group, high glucose induced expression levels of IL‐6, IL‐10 and p‐NF‐κBp65 in AML12 cells (Figure 6A‐D). FGF1 treatment suppressed high glucose–induced up‐regulation of IL‐6 and p‐NF‐κBp65 and further promoted the expression of IL‐10 in AML12 cells (Figure 6A‐D). However, co‐treating with D‐ribose reversed the protective role of FGF1 on AML12 cells under high glucose condition (Figure 6A‐D).

## 4 DISCUSSION

As the liver is an important metabolic organ, DMLD causes a series of damage to patients.^2^ Moreover, chronic liver injury will also aggravate diabetes.^26^ Thus, it is very important to find the therapeutic target and develop the effective drug for DMLD. It has been reported that RAGE is involved in the fibrosis during NASH and T2DM.^27^ In our current study, we observed that diabetes significantly causes liver dysfunction via triggering steatosis and fibrosis. FGF1 treatment reduces apoptosis and inflammation in the liver, and consequently ameliorates diabetes‐induced liver injury though suppressing RAGE pathway.

FGF1 was regarded as a potential weapon for treating diabetes because of its excellent blood glucose control and insulin sensitization effects.^13^, ^28^, ^29^ According to the recent studies, FGF1 not only improves insulin resistance through regulating JNK pathway,^16^ but also subsequently performs other functions to treat diabetic complications. It has been reported that FGF1 ameliorates diabetic nephropathy through exerting its anti‐inflammatory function and suppressing the cellular stress in kidney.^15^, ^30^ Moreover, exogenous FGF1 treatment can reverse diabetes‐induced suppression of brain‐derived neurotrophic factor (BDNF) level and elevated neuronal apoptosis, then ameliorate cognitive decline.^31^ More importantly, it was previously reported that rFGF1 stimulates liver fat metabolism and effectively ameliorates liver inflammation and damage in ob/ob mice.^32^ Our previous study has also demonstrated that FGF1 treatment ameliorates DMLD by reducing cellular stress and restoring autophagy.^17^ Consistent with these findings, our current study has confirmed the protective role of FGF1 on DMLD (Figure 1). Moreover, a novel finding of our research was that FGF1 exerts its anti‐apoptotic and anti‐inflammatory function to protect the liver via suppressing RAGE pathway. These results will provide a new idea for the treatment of DMLD.

Among many potential pathogenesis, excessive inflammation is an important cause factor for series of diabetes‐mediated complications. Many studies have shown that obesity and T2DM are considered as inflammatory diseases.^33^, ^34^ NF‐κBp65 is a key transcription factor for the expressions of many pro‐inflammatory genes.^35^ Diabetes activates NF‐κBp65 activity in a variety of cell types, and FGF1 treatment reverses this effect and reduces inflammation.^15^ In our current study, the elevated pro‐inflammatory factors were observed in the serum and liver tissue of db/db mice. FGF1 treatment reversed them. However, we surprisingly observed that anti‐inflammatory (IL‐10) level of db/db mice was not lower than that of db/m mice. Additionally, we observed the same result in AML12 cells. These results suggest that under hyperglycaemia condition, enhanced IL‐10 level is an adaptive response to resist the body's inflammation, and FGF1 administration further promotes the secretion of anti‐inflammatory cytokines (IL‐10). Taken together, our and the others’ studies suggest that the chronic inflammatory control is the critical mechanism underlying FGF1 ameliorating diabetes‐associated complications.

RAGE plays an important role in DMLD. According to the Ali Dehnad et al study, RAGE, a pro‐inflammatory receptor, was induced in patients with NASH and diabetes.^27^ Consistent with the previous studies, we found that the RAGE expression in the liver of db/db mice was significantly higher than that in the liver of db/m mice, and it was reversed by FGF1 treatment. According to the previous studies, the RAGE/NF‐κBp65 pathway is related to inflammation under diabetes environment.^36^ Our animal experiments had also found that diabetes triggers elevated RAGE level and activates NF‐κBp65 activity in the liver. In order to further verify our hypothesis, D‐ribose, a RAGE agonist, was used to treat AML12 cells under high glucose condition. We found that RAGE is essential for high glucose–mediated hepatocyte inflammation by regulating the activity of NF‐κBp65 on AML12 cells. Meanwhile, FGF1 ameliorates high glucose–induced elevated inflammation and apoptosis via suppressing RAGE pathway. These results suggest that RAGE/NF‐κBp65–mediated inflammation is a critical mechanism during DMLD, and FGF1 reduces inflammation and then ameliorates high glucose‐induced excessive apoptosis via inhibiting RAGE expression. However, whether FGF1 directly or indirectly regulates the RAGE pathway remains to be further studied. In future, we will further investigate how FGF1 affects RAGE expression.

Diabetes significantly induces excessive apoptosis, which is a major molecular mechanism for diabetes‐associated complications. There are many factors that promote apoptosis, such as inflammatory response, endoplasmic reticulum(ER) stress, autophagy and other factors.^19^, ^37^, ^38^ In our current study, we also found that hepatocyte apoptosis is accompanied by an increase in RAGE level. Additionally, elevated RAGE contributed to cell apoptosis in the liver under high glucose condition. FGF1 treatment reversed them (Figure 5). These results demonstrate that FGF1 blocks diabetes‐induced excessive hepatocyte apoptosis in the liver through regulating RAGE expression. However, whether the excessive inflammatory response that mediated by RAGE/NF‐κBp65 may cause hepatocyte apoptosis, or other RAGE‐dependent pathway is involved in hepatocyte apoptosis, or the synergistic effect of the two factors, needs to be further studied. This is the limitation of our current study.

Based on our current experimental results, it is indicated that RAGE is a key target during FGF1 treatment of DMLD. It has been reported that olmesartan attenuates type 2 diabetes‐associated liver injury via suppressing AGE/RAGE/JNK pathway.^39^ Additionally, RAGE/MAPK/ERK is suppressed to reduce cellular stress and then apoptosis.^40^, ^41^ Furthermore, RAGE can regulate the PI3K/AKT/mTOR pathway and involve in the development of disease.^5^, ^42^ Thus, we further speculate that the effect of FGF1 in the liver is mediated by RAGE‐related signals, which needs to be further verified.

**FIGURE 7:**
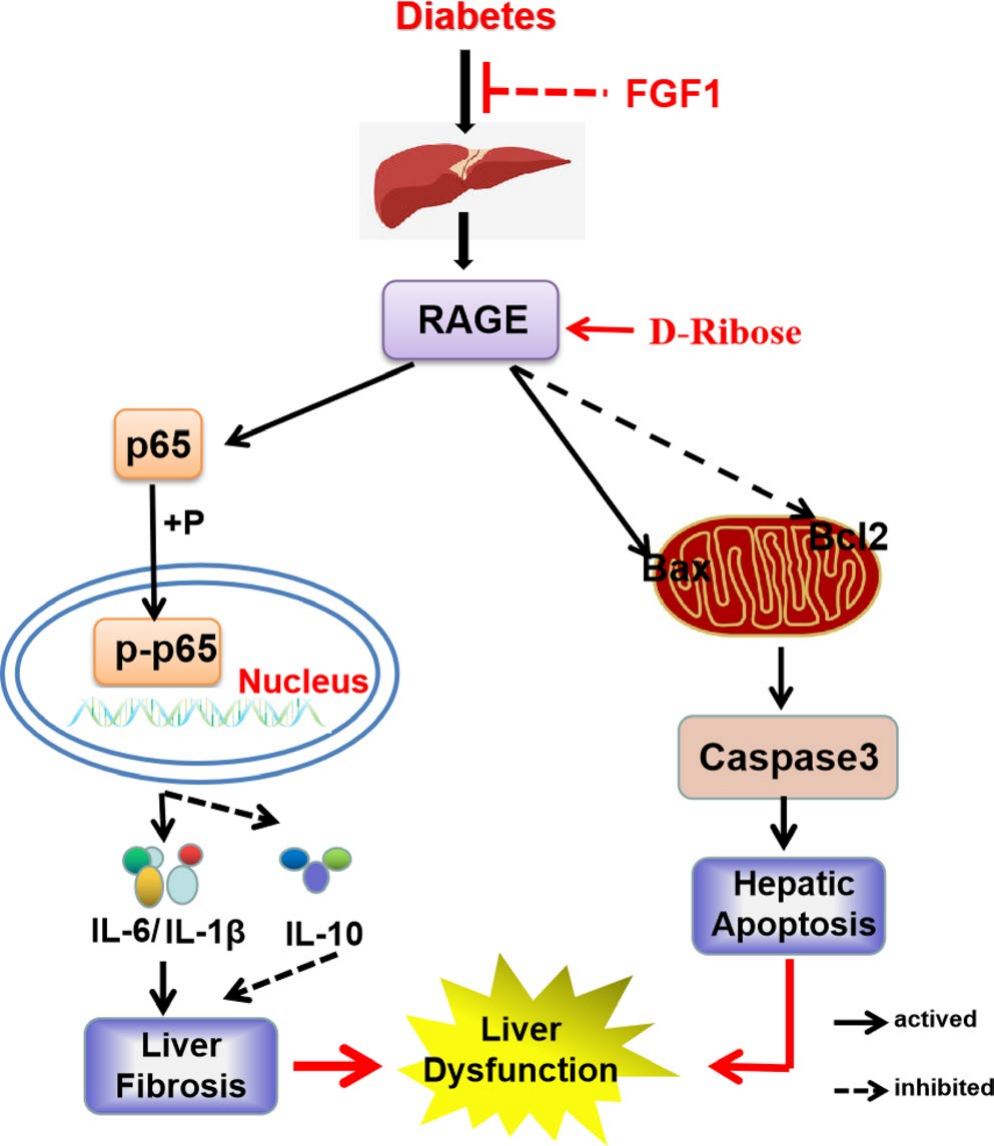
Schematic showing the role of RAGE during FGF1 ameliorating diabetes‐induced liver dysfunction. Diabetes induces liver inflammation through the RAGE/NF‐κBp65 pathway, thereby promoting fibrosis and steatosis in the liver. Meanwhile, diabetes triggers the RAGE pathway and then promotes Bax expression and inhibits Bcl2 expression, which subsequently contributes to hepatic apoptosis. FGF1 treatment ameliorates the effect of diabetes on excessive inflammation and apoptosis. More importantly, D‐ribose (a RAGE agonist) treatment significantly reverses the protective role of FGF1 on diabetes‐induced liver inflammation and apoptosis, suggesting that RAGE may be a potential therapeutic target during FGF1 treatment of diabetes‐induced liver injury

In summary, our current study found that diabetes significantly triggers liver steatosis and fibrosis and consequently leads to liver dysfunction with excessive inflammation and hepatocyte apoptosis. Mechanism studies showed that diabetes remarkably induces the expression of RAGE in the liver, which not only causes excessive cell apoptosis, but also triggers an inflammatory response through the RAGE/NF‐κBp65 signalling pathway. FGF1 can effectively prevent diabetes‐induced elevated apoptosis and inflammation, and consequently ameliorate liver injury though reducing RAGE expression and inhibiting the RAGE/NF‐κBp65 pathway (Figure 7). These studies indicate that RAGE may be an effective and potential target for FGF1 treatment of DMLD.

## CONFLICT OF INTEREST

The authors confirm that there are no conflicts of interest.

## AUTHOR CONTRIBUTIONS

**Peipei Zheng:** Data curation (equal). **Zonghao Tang:** Data curation (equal). **Jun Xiong:** Data curation (equal). **Beini Wang:** Data curation (supporting). **Jingyu Xu:** Data curation (supporting). **Lulu Chen:** Data curation (supporting). **Shufang Cai:** Data curation (supporting). **Chengbiao Wu:** Funding acquisition (supporting); Writing‐review & editing (supporting). **Libing Ye:** Formal analysis (supporting); Writing‐review & editing (supporting). **ke xu:** Data curation (supporting); Funding acquisition (supporting). **Zimiao Chen:** Funding acquisition (equal); Project administration (equal). **Yanqing Wu:** Data curation (equal); Funding acquisition (equal); Project administration (equal); Writing‐review & editing (equal). **Jian Xiao:** Data curation (equal); Project administration (equal); Writing‐review & editing (equal).

## Data Availability

The data that support the findings of this study are available from the corresponding author upon reasonable request.
